# Implementing IG Best Practices in Community-Based Settings: A Pre-Implementation Study

**DOI:** 10.1093/geroni/igaa057.2605

**Published:** 2020-12-16

**Authors:** Lisa Juckett, Shannon Jarrott, Jill Naar, Rachel Scivano, Alicia Bunger

**Affiliations:** 1 The Ohio State University, Columbus, Ohio, United States; 2 Appalachian State University, Boone, North Carolina, United States; 3 Ohio State University, Columbus, Ohio, United States

## Abstract

Programs that intentionally engage unrelated young and old persons often lead to mutual benefits; however, specific implementation strategies that support the use of evidence-based intergenerational programming in community settings are understudied. With strong demand for training resources among intergenerational program providers, this pilot study examined how a multifaceted training strategy facilitated the implementation of 14 distinct evidence-based intergenerational best practices. Intergenerational programming was implemented with nine staff from two small community sites using three implementation strategies including educational meetings, ongoing consultation, and routine practice reminders. Observational analysis of video recorded intergenerational program sessions indicated that staff adopted an average of 81.7% of intergenerational best practices suggesting the feasibility of implementing IG in community settings. Findings yield valuable insight that can inform training refinements, and selection of strategies for improving implementation. Next steps include aligning specific practices with participant outcomes.

